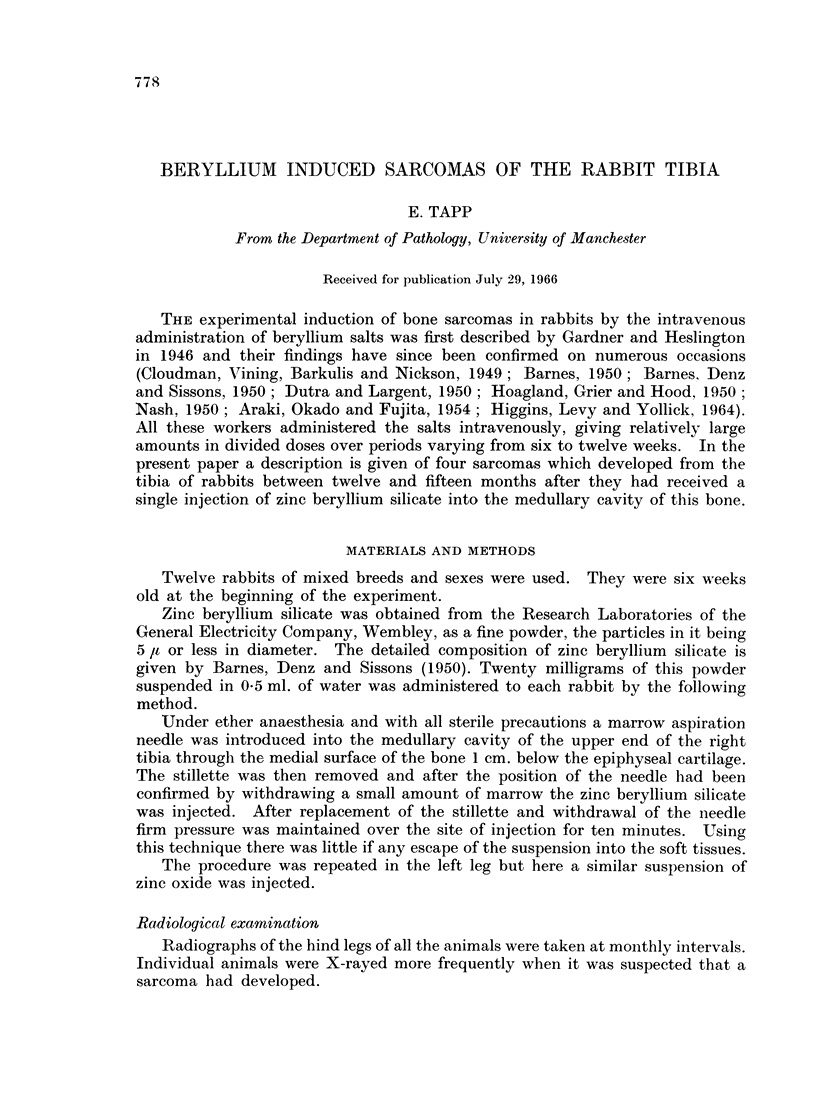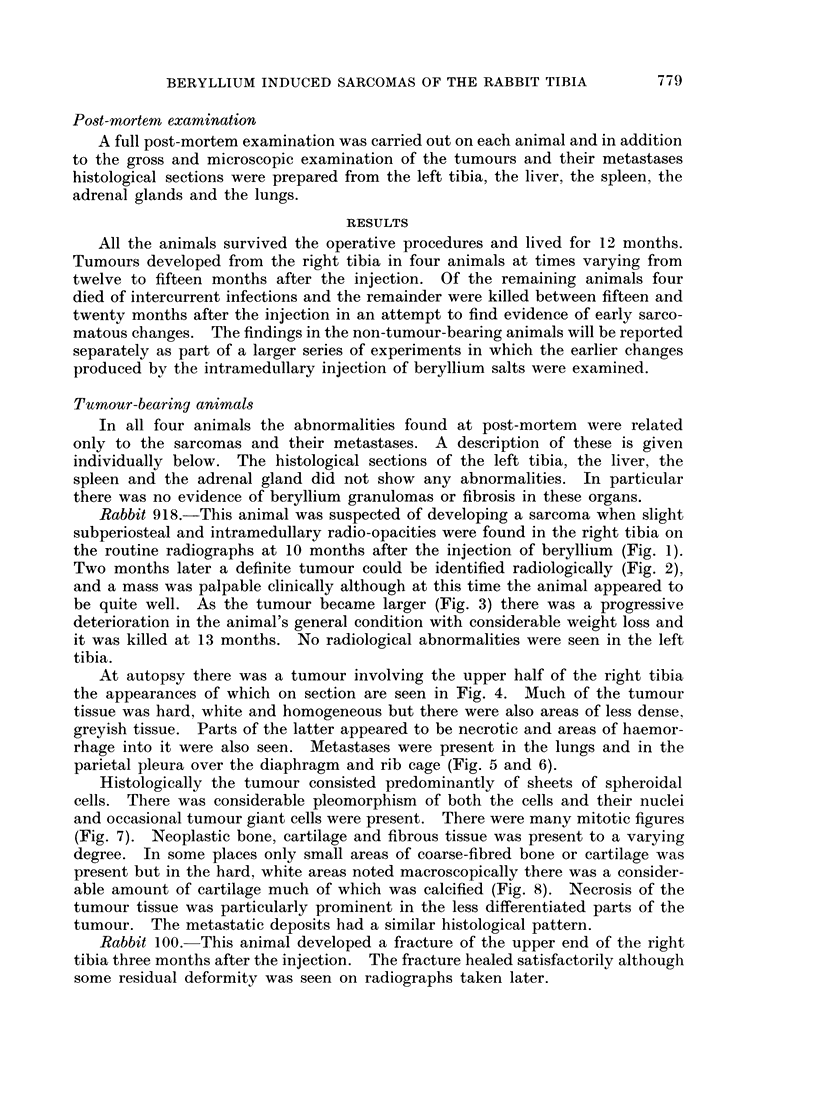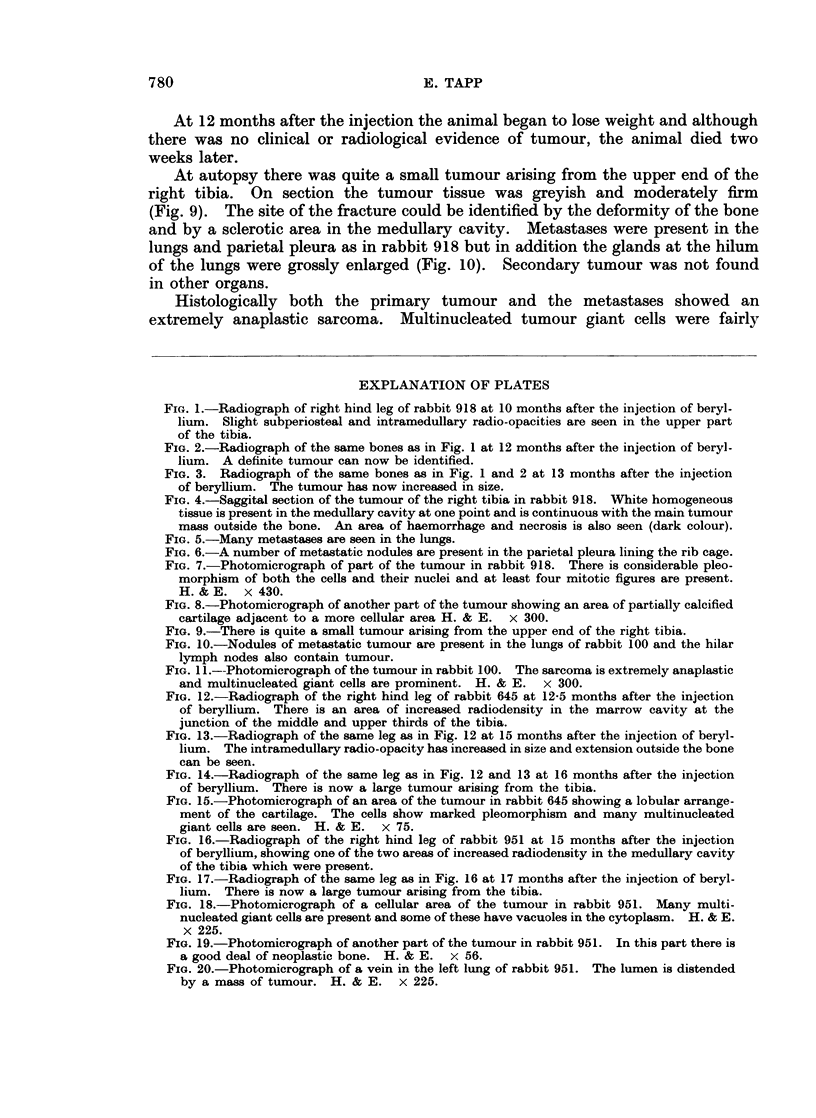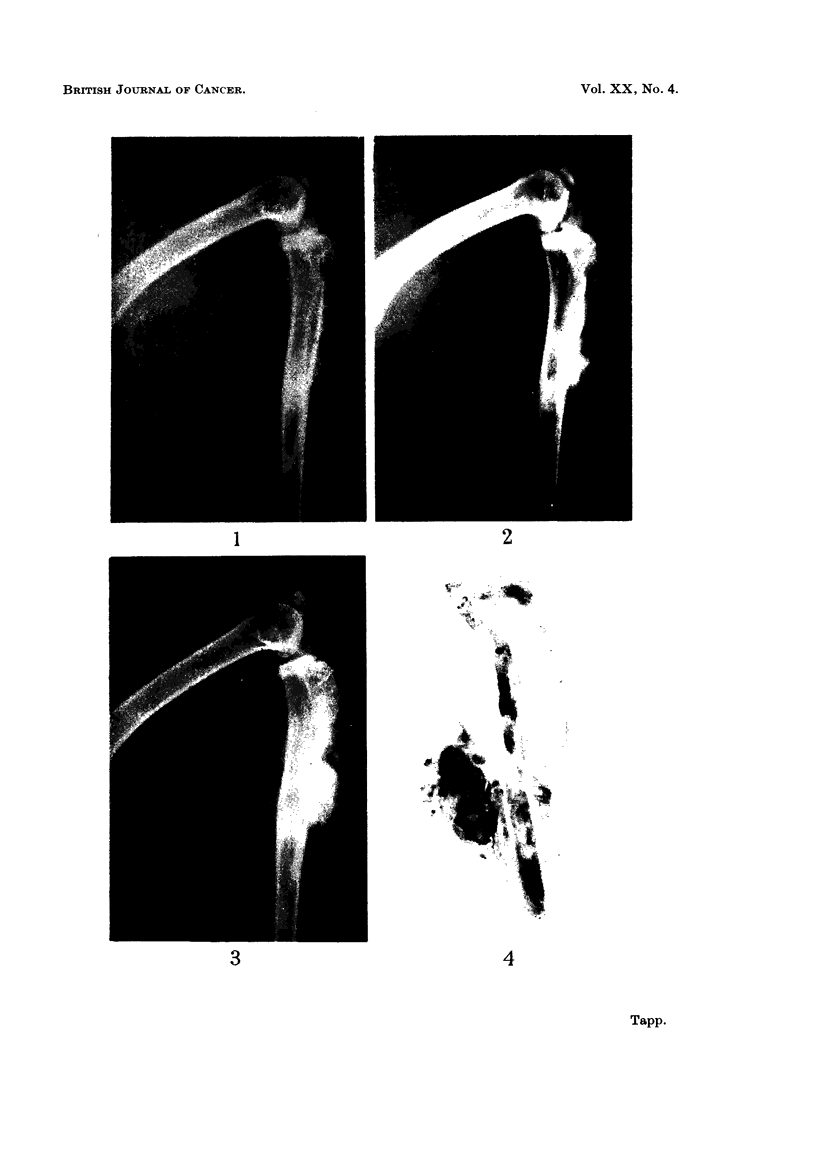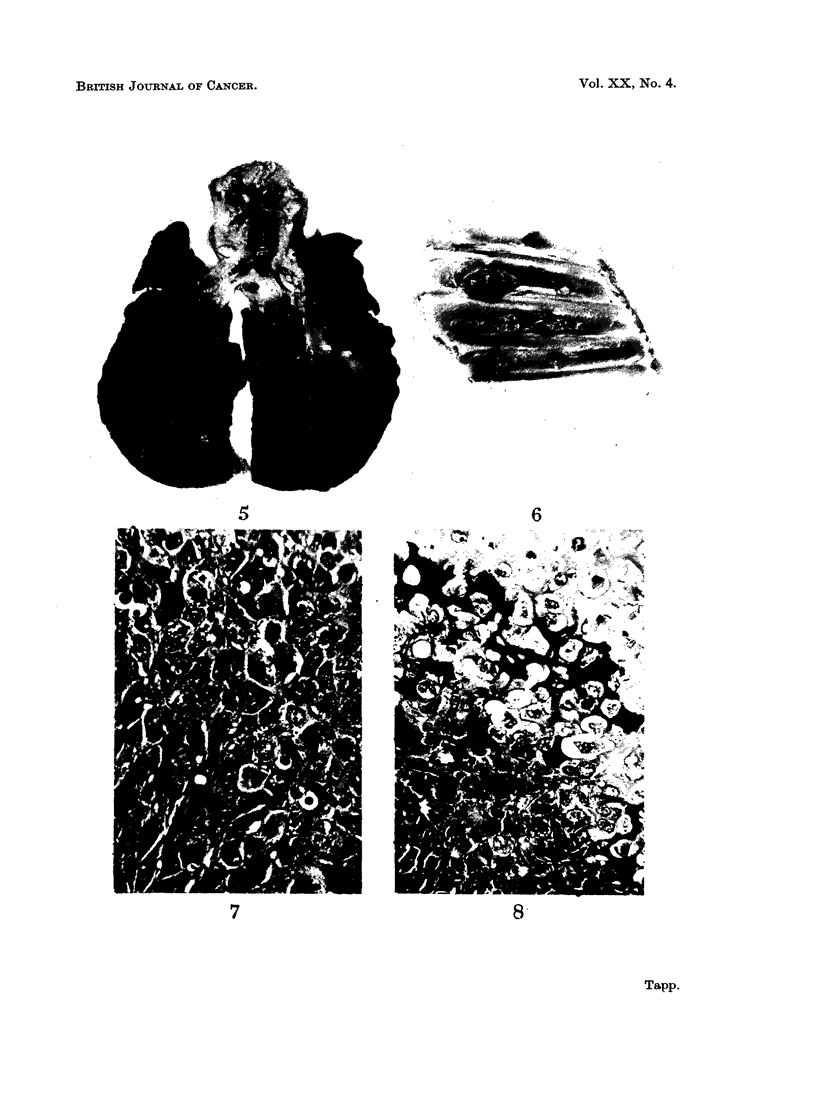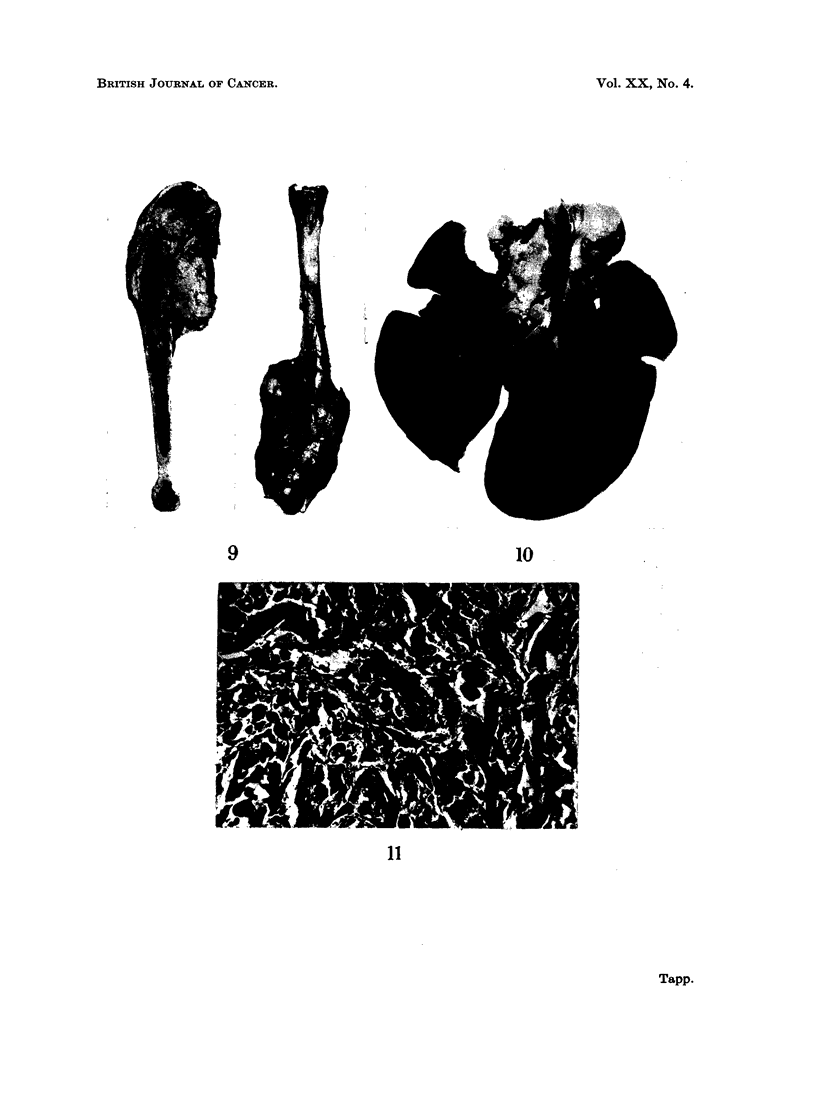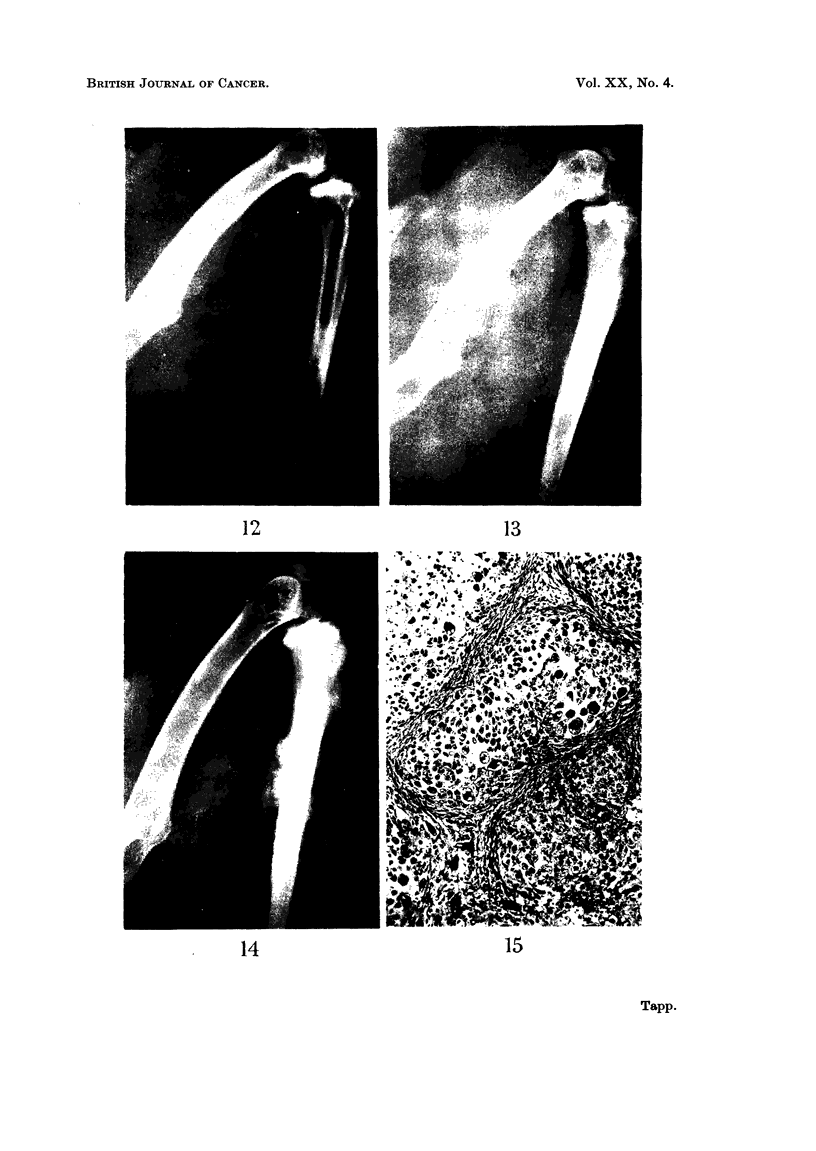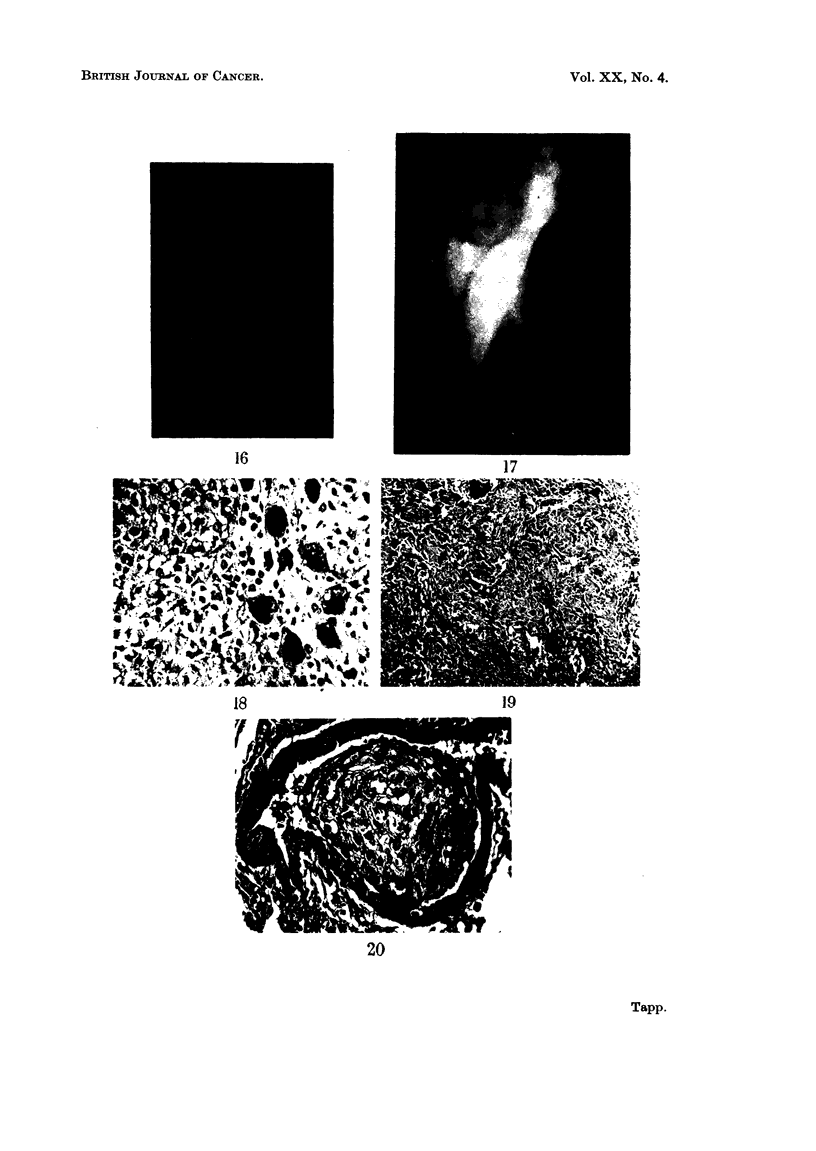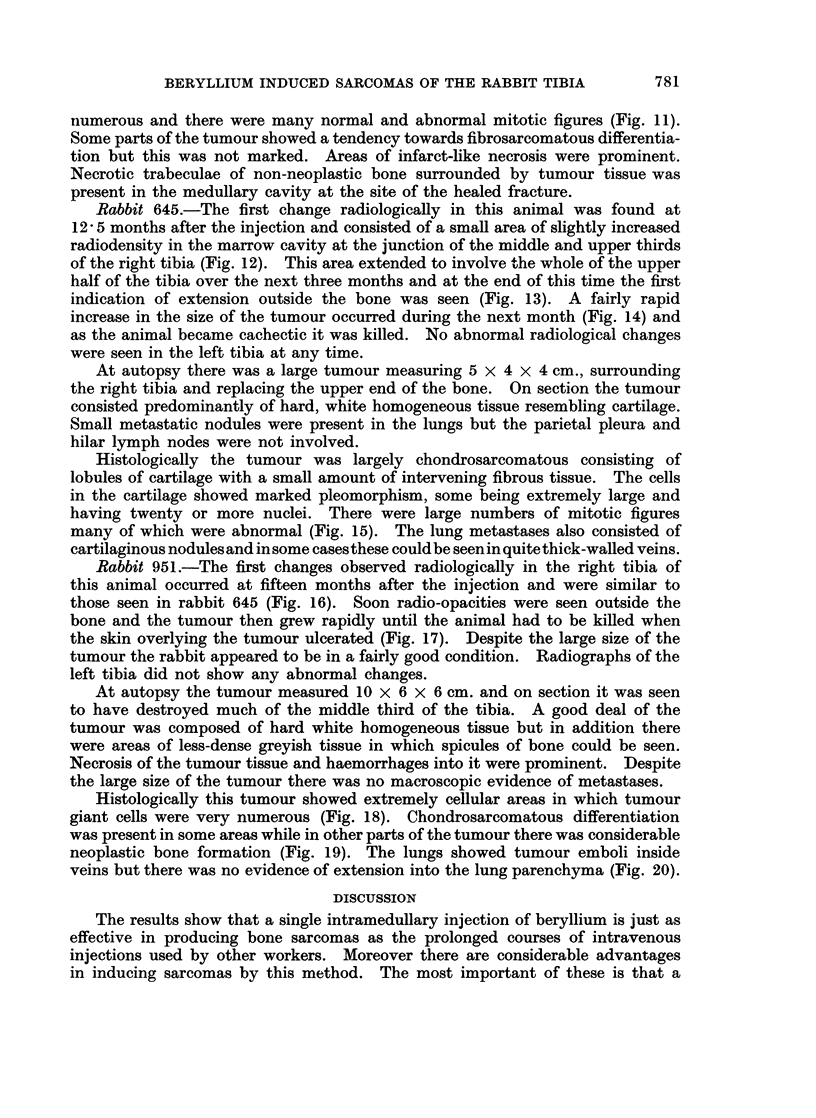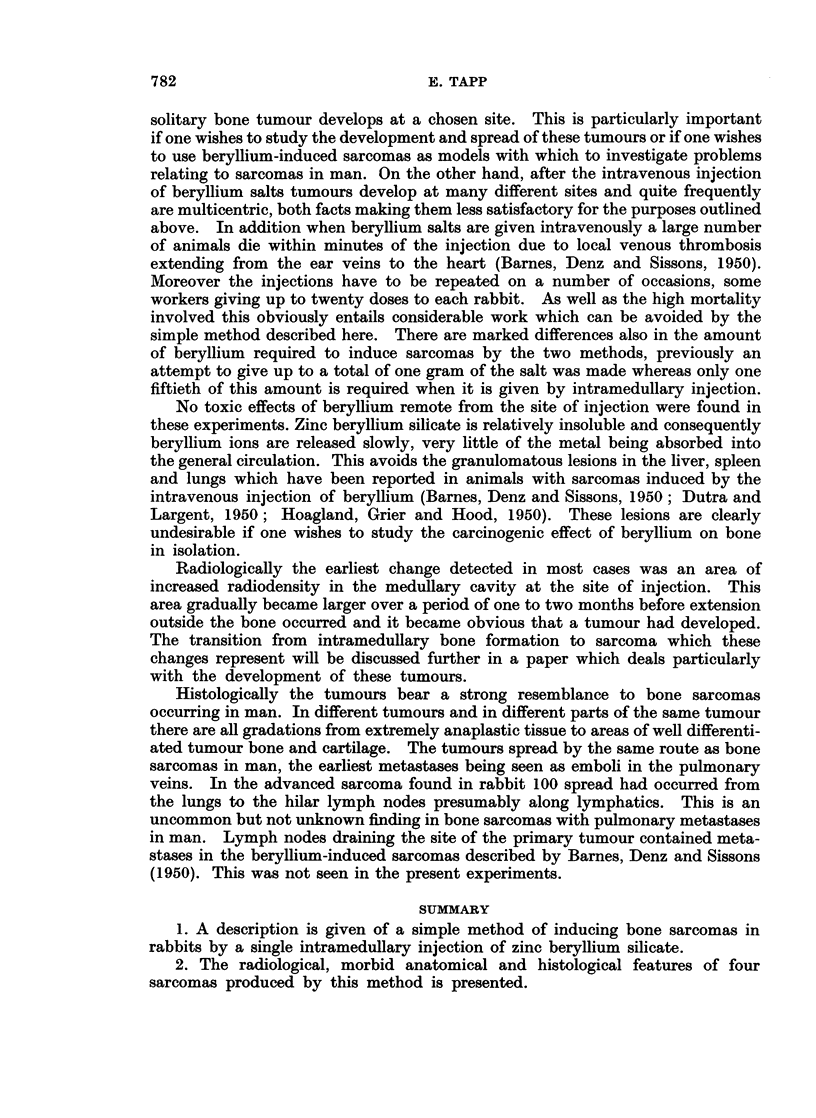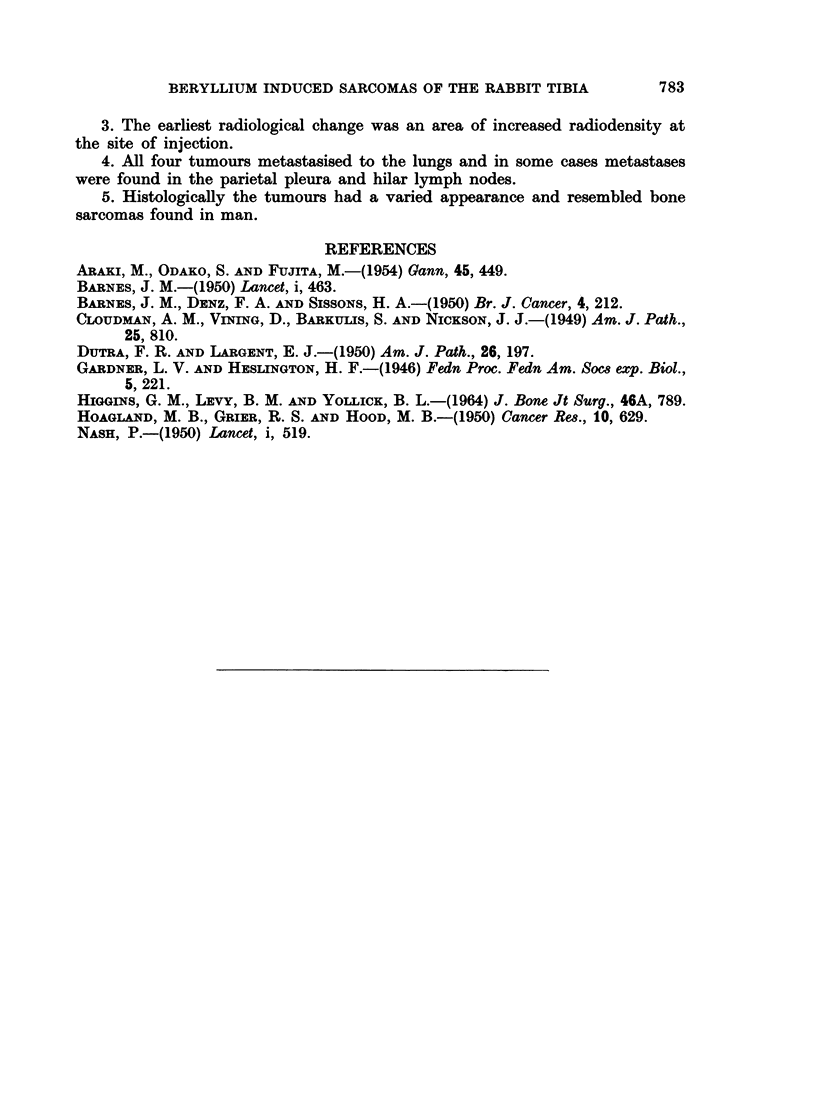# Beryllium induced sarcomas of the rabbit tibia.

**DOI:** 10.1038/bjc.1966.89

**Published:** 1966-12

**Authors:** E. Tapp

## Abstract

**Images:**


### BERYLLIUM INDUCED SARCOMAS OF THE RABBIT TIBIA

E. TAPP

*From the Department of Pathology, University of Manchester*

Received for publication July 29, 1966

THE experimental induction of bone sarcomas in rabbits by the intravenous administration of beryllium salts was first described by Gardner and Heslington in 1946 and their findings have since been confirmed on numerous occasions (Cloudman, Vining, Barkulis and Nickson, 1949 ; Barnes, 1950 ; Barnes, Denz and Sissons, 1950 ; Dutra and Largent, 1950 ; Hoagland, Grier and Hood, 1950 ; Nash, 1950 ; Araki, Okado and Fujita, 1954 ; Higgins, Levy and Yollick, 1964). All these workers administered the salts intravenously, giving relatively large amounts in divided doses over periods varying from six to twelve weeks. In the present paper a description is given of four sarcomas which developed from the tibia of rabbits between twelve and fifteen months after they had received a single injection of zinc beryllium silicate into the medullary cavity of this bone.

### MATERIALS AND METHODS

Twelve rabbits of mixed breeds and sexes were used. They were six weeks old at the beginning of the experiment.

Zinc beryllium silicate was obtained from the Research Laboratories of the General Electricity Company, Wembley, as a fine powder, the particles in it being 5 $\mu$ or less in diameter. The detailed composition of zinc beryllium silicate is given by Barnes, Denz and Sissons (1950). Twenty milligrams of this powder suspended in 0·5 ml. of water was administered to each rabbit by the following method.

Under ether anaesthesia and with all sterile precautions a marrow aspiration needle was introduced into the medullary cavity of the upper end of the right tibia through the medial surface of the bone 1 cm. below the epiphyseal cartilage. The stillette was then removed and after the position of the needle had been confirmed by withdrawing a small amount of marrow the zinc beryllium silicate was injected. After replacement of the stillette and withdrawal of the needle firm pressure was maintained over the site of injection for ten minutes. Using this technique there was little if any escape of the suspension into the soft tissues.

The procedure was repeated in the left leg but here a similar suspension of zinc oxide was injected.

### *Radiological examination*

Radiographs of the hind legs of all the animals were taken at monthly intervals. Individual animals were X-rayed more frequently when it was suspected that a sarcoma had developed.

*Post-mortem examination*

A full post-mortem examination was carried out on each animal and in addition to the gross and microscopic examination of the tumours and their metastases histological sections were prepared from the left tibia, the liver, the spleen, the adrenal glands and the lungs.

### RESULTS

All the animals survived the operative procedures and lived for 12 months. Tumours developed from the right tibia in four animals at times varying from twelve to fifteen months after the injection. Of the remaining animals four died of intercurrent infections and the remainder were killed between fifteen and twenty months after the injection in an attempt to find evidence of early sarcomatous changes. The findings in the non-tumour-bearing animals will be reported separately as part of a larger series of experiments in which the earlier changes produced by the intramedullary injection of beryllium salts were examined.

*Tumour-bearing animals*

In all four animals the abnormalities found at post-mortem were related only to the sarcomas and their metastases. A description of these is given individually below. The histological sections of the left tibia, the liver, the spleen and the adrenal gland did not show any abnormalities. In particular there was no evidence of beryllium granulomas or fibrosis in these organs.

*Rabbit* 918.—This animal was suspected of developing a sarcoma when slight subperiosteal and intramedullary radio-opacities were found in the right tibia on the routine radiographs at 10 months after the injection of beryllium (Fig. 1). Two months later a definite tumour could be identified radiologically (Fig. 2), and a mass was palpable clinically although at this time the animal appeared to be quite well. As the tumour became larger (Fig. 3) there was a progressive deterioration in the animal's general condition with considerable weight loss and it was killed at 13 months. No radiological abnormalities were seen in the left tibia.

At autopsy there was a tumour involving the upper half of the right tibia the appearances of which on section are seen in Fig. 4. Much of the tumour tissue was hard, white and homogeneous but there were also areas of less dense, greyish tissue. Parts of the latter appeared to be necrotic and areas of haemorrhage into it were also seen. Metastases were present in the lungs and in the parietal pleura over the diaphragm and rib cage (Fig. 5 and 6).

Histologically the tumour consisted predominantly of sheets of spheroidal cells. There was considerable pleomorphism of both the cells and their nuclei and occasional tumour giant cells were present. There were many mitotic figures (Fig. 7). Neoplastic bone, cartilage and fibrous tissue was present to a varying degree. In some places only small areas of coarse-fibred bone or cartilage was present but in the hard, white areas noted macroscopically there was a considerable amount of cartilage much of which was calcified (Fig. 8). Necrosis of the tumour tissue was particularly prominent in the less differentiated parts of the tumour. The metastatic deposits had a similar histological pattern.

*Rabbit* 100.—This animal developed a fracture of the upper end of the right tibia three months after the injection. The fracture healed satisfactorily although some residual deformity was seen on radiographs taken later.

At 12 months after the injection the animal began to lose weight and although there was no clinical or radiological evidence of tumour, the animal died two weeks later.

At autopsy there was quite a small tumour arising from the upper end of the right tibia. On section the tumour tissue was greyish and moderately firm (Fig. 9). The site of the fracture could be identified by the deformity of the bone and by a sclerotic area in the medullary cavity. Metastases were present in the lungs and parietal pleura as in rabbit 918 but in addition the glands at the hilum of the lungs were grossly enlarged (Fig. 10). Secondary tumour was not found in other organs.

Histologically both the primary tumour and the metastases showed an extremely anaplastic sarcoma. Multinucleated tumour giant cells were fairly

---

### EXPLANATION OF PLATES

Fig. 1.—Radiograph of right hind leg of rabbit 918 at 10 months after the injection of beryllium. Slight subperiosteal and intramedullary radio-opacities are seen in the upper part of the tibia.

Fig. 2.—Radiograph of the same bones as in Fig. 1 at 12 months after the injection of beryllium. A definite tumour can now be identified.

Fig. 3. Radiograph of the same bones as in Fig. 1 and 2 at 13 months after the injection of beryllium. The tumour has now increased in size.

Fig. 4.—Saggital section of the tumour of the right tibia in rabbit 918. White homogeneous tissue is present in the medullary cavity at one point and is continuous with the main tumour mass outside the bone. An area of haemorrhage and necrosis is also seen (dark colour).

Fig. 5.—Many metastases are seen in the lungs.

Fig. 6.—A number of metastatic nodules are present in the parietal pleura lining the rib cage.

Fig. 7.—Photomicrograph of part of the tumour in rabbit 918. There is considerable pleomorphism of both the cells and their nuclei and at least four mitotic figures are present. H. & E. × 430.

Fig. 8.—Photomicrograph of another part of the tumour showing an area of partially calcified cartilage adjacent to a more cellular area H. & E. × 300.

Fig. 9.—There is quite a small tumour arising from the upper end of the right tibia.

Fig. 10.—Nodules of metastatic tumour are present in the lungs of rabbit 100 and the hilar lymph nodes also contain tumour.

Fig. 11.—Photomicrograph of the tumour in rabbit 100. The sarcoma is extremely anaplastic and multinucleated giant cells are prominent. H. & E. × 300.

Fig. 12.—Radiograph of the right hind leg of rabbit 645 at 12·5 months after the injection of beryllium. There is an area of increased radiodensity in the marrow cavity at the junction of the middle and upper thirds of the tibia.

Fig. 13.—Radiograph of the same leg as in Fig. 12 at 15 months after the injection of beryllium. The intramedullary radio-opacity has increased in size and extension outside the bone can be seen.

Fig. 14.—Radiograph of the same leg as in Fig. 12 and 13 at 16 months after the injection of beryllium. There is now a large tumour arising from the tibia.

Fig. 15.—Photomicrograph of an area of the tumour in rabbit 645 showing a lobular arrangement of the cartilage. The cells show marked pleomorphism and many multinucleated giant cells are seen. H. & E. × 75.

Fig. 16.—Radiograph of the right hind leg of rabbit 951 at 15 months after the injection of beryllium, showing one of the two areas of increased radiodensity in the medullary cavity of the tibia which were present.

Fig. 17.—Radiograph of the same leg as in Fig. 16 at 17 months after the injection of beryllium. There is now a large tumour arising from the tibia.

Fig. 18.—Photomicrograph of a cellular area of the tumour in rabbit 951. Many multinucleated giant cells are present and some of these have vacuoles in the cytoplasm. H. & E. × 225.

Fig. 19.—Photomicrograph of another part of the tumour in rabbit 951. In this part there is a good deal of neoplastic bone. H. & E. × 56.

Fig. 20.—Photomicrograph of a vein in the left lung of rabbit 951. The lumen is distended by a mass of tumour. H. & E. × 225.

1

2

3

4

Tapp.

5

6

7

8

Tapp.

9

10

11

Tapp.

12

13

14

15

Tapp.

16

17

18

19

20

numerous and there were many normal and abnormal mitotic figures (Fig. 11). Some parts of the tumour showed a tendency towards fibrosarcomatous differentiation but this was not marked. Areas of infarct-like necrosis were prominent. Necrotic trabeculae of non-neoplastic bone surrounded by tumour tissue was present in the medullary cavity at the site of the healed fracture.

*Rabbit* 645.—The first change radiologically in this animal was found at 12·5 months after the injection and consisted of a small area of slightly increased radiodensity in the marrow cavity at the junction of the middle and upper thirds of the right tibia (Fig. 12). This area extended to involve the whole of the upper half of the tibia over the next three months and at the end of this time the first indication of extension outside the bone was seen (Fig. 13). A fairly rapid increase in the size of the tumour occurred during the next month (Fig. 14) and as the animal became cachectic it was killed. No abnormal radiological changes were seen in the left tibia at any time.

At autopsy there was a large tumour measuring 5 × 4 × 4 cm., surrounding the right tibia and replacing the upper end of the bone. On section the tumour consisted predominantly of hard, white homogeneous tissue resembling cartilage. Small metastatic nodules were present in the lungs but the parietal pleura and hilar lymph nodes were not involved.

Histologically the tumour was largely chondrosarcomatous consisting of lobules of cartilage with a small amount of intervening fibrous tissue. The cells in the cartilage showed marked pleomorphism, some being extremely large and having twenty or more nuclei. There were large numbers of mitotic figures many of which were abnormal (Fig. 15). The lung metastases also consisted of cartilaginous nodules and in some cases these could be seen in quite thick-walled veins.

*Rabbit* 951.—The first changes observed radiologically in the right tibia of this animal occurred at fifteen months after the injection and were similar to those seen in rabbit 645 (Fig. 16). Soon radio-opacities were seen outside the bone and the tumour then grew rapidly until the animal had to be killed when the skin overlying the tumour ulcerated (Fig. 17). Despite the large size of the tumour the rabbit appeared to be in a fairly good condition. Radiographs of the left tibia did not show any abnormal changes.

At autopsy the tumour measured 10 × 6 × 6 cm. and on section it was seen to have destroyed much of the middle third of the tibia. A good deal of the tumour was composed of hard white homogeneous tissue but in addition there were areas of less-dense greyish tissue in which spicules of bone could be seen. Necrosis of the tumour tissue and haemorrhages into it were prominent. Despite the large size of the tumour there was no macroscopic evidence of metastases.

Histologically this tumour showed extremely cellular areas in which tumour giant cells were very numerous (Fig. 18). Chondrosarcomatous differentiation was present in some areas while in other parts of the tumour there was considerable neoplastic bone formation (Fig. 19). The lungs showed tumour emboli inside veins but there was no evidence of extension into the lung parenchyma (Fig. 20).

### DISCUSSION

The results show that a single intramedullary injection of beryllium is just as effective in producing bone sarcomas as the prolonged courses of intravenous injections used by other workers. Moreover there are considerable advantages in inducing sarcomas by this method. The most important of these is that a solitary bone tumour develops at a chosen site. This is particularly important if one wishes to study the development and spread of these tumours or if one wishes to use beryllium-induced sarcomas as models with which to investigate problems relating to sarcomas in man. On the other hand, after the intravenous injection of beryllium salts tumours develop at many different sites and quite frequently are multicentric, both facts making them less satisfactory for the purposes outlined above. In addition when beryllium salts are given intravenously a large number of animals die within minutes of the injection due to local venous thrombosis extending from the ear veins to the heart (Barnes, Denz and Sissons, 1950). Moreover the injections have to be repeated on a number of occasions, some workers giving up to twenty doses to each rabbit. As well as the high mortality involved this obviously entails considerable work which can be avoided by the simple method described here. There are marked differences also in the amount of beryllium required to induce sarcomas by the two methods, previously an attempt to give up to a total of one gram of the salt was made whereas only one fiftieth of this amount is required when it is given by intramedullary injection.

No toxic effects of beryllium remote from the site of injection were found in these experiments. Zinc beryllium silicate is relatively insoluble and consequently beryllium ions are released slowly, very little of the metal being absorbed into the general circulation. This avoids the granulomatous lesions in the liver, spleen and lungs which have been reported in animals with sarcomas induced by the intravenous injection of beryllium (Barnes, Denz and Sissons, 1950 ; Dutra and Largent, 1950 ; Hoagland, Grier and Hood, 1950). These lesions are clearly undesirable if one wishes to study the carcinogenic effect of beryllium on bone in isolation.

Radiologically the earliest change detected in most cases was an area of increased radiodensity in the medullary cavity at the site of injection. This area gradually became larger over a period of one to two months before extension outside the bone occurred and it became obvious that a tumour had developed. The transition from intramedullary bone formation to sarcoma which these changes represent will be discussed further in a paper which deals particularly with the development of these tumours.

Histologically the tumours bear a strong resemblance to bone sarcomas occurring in man. In different tumours and in different parts of the same tumour there are all gradations from extremely anaplastic tissue to areas of well differentiated tumour bone and cartilage. The tumours spread by the same route as bone sarcomas in man, the earliest metastases being seen as emboli in the pulmonary veins. In the advanced sarcoma found in rabbit 100 spread had occurred from the lungs to the hilar lymph nodes presumably along lymphatics. This is an uncommon but not unknown finding in bone sarcomas with pulmonary metastases in man. Lymph nodes draining the site of the primary tumour contained metastases in the beryllium-induced sarcomas described by Barnes, Denz and Sissons (1950). This was not seen in the present experiments.

### SUMMARY

1. A description is given of a simple method of inducing bone sarcomas in rabbits by a single intramedullary injection of zinc beryllium silicate.

2. The radiological, morbid anatomical and histological features of four sarcomas produced by this method is presented.

3. The earliest radiological change was an area of increased radiodensity at the site of injection.

4. All four tumours metastasised to the lungs and in some cases metastases were found in the parietal pleura and hilar lymph nodes.

5. Histologically the tumours had a varied appearance and resembled bone sarcomas found in man.

### REFERENCES

ARAKI, M., ODAKO, S. AND FUJITA, M.—(1954) *Gann*, **45**, 449.

BARNES, J. M.—(1950) *Lancet*, i, 463.

BARNES, J. M., DENZ, F. A. AND SISSONS, H. A.—(1950) *Br. J. Cancer*, **4**, 212.

CLOUDMAN, A. M., VINING, D., BARKULIS, S. AND NICKSON, J. J.—(1949) *Am. J. Path.*, **25**, 810.

DUTRA, F. R. AND LARGENT, E. J.—(1950) *Am. J. Path.*, **26**, 197.

GARDNER, L. V. AND HESLINGTON, H. F.—(1946) *Fedn Proc. Fedn Am. Socs exp. Biol.*, **5**, 221.

HIGGINS, G. M., LEVY, B. M. AND YOLLICK, B. L.—(1964) *J. Bone Jt Surg.*, **46**A, 789.

HOAGLAND, M. B., GRIER, R. S. AND HOOD, M. B.—(1950) *Cancer Res.*, **10**, 629.

NASH, P.—(1950) *Lancet*, i, 519.